# Shrub Growth Improves Morphological Features of Nebkhas: A Case Study of *Nitraria tangutorum* in the Tengger Desert

**DOI:** 10.3390/plants13050624

**Published:** 2024-02-24

**Authors:** Long Cheng, Bo Wu, Yingjun Pang, Xiaohong Jia

**Affiliations:** 1 Academy of Agriculture and Forestry Sciences, Qinghai University, Xining 810016, China; 2 Institute of Ecological Conservation and Restoration, Chinese Academy of Forestry, Beijing 100091, China; 3 Institute of Desertification Studies, Chinese Academy of Forestry, Beijing 100091, China; 4 Key Laboratory of Desert Ecosystem and Global Change, State Administration of Forestry and Grassland, Beijing 100091, China

**Keywords:** nebkha morphology, arid desert, nebkha development, sand burial, spatial heterogeneity

## Abstract

To understand the role of shrubs in nebkha development, a comparative analysis of nebkha morphology and shrub features was conducted in two different habitats at the southeast margin of the Tengger Desert, Northern China. Morphometric variables of 184 *Nitraria tangutorum* nebkhas were measured in a semi-fixed lake-basin lowland site (site 1, *n* = 102) and a salinized fixed sand site (site 2, *n* = 82). Mean length, width, projected area, and accumulated sand volume were all greater in nebkhas in site 1 than in site 2 (*p* < 0.05); however, mean height (i.e., sand burial depth) did not differ significantly in nebkhas between the two sites (*p* > 0.05). The larger nebkha volume in site 1 relative to site 2 (mean, 88.19 m^3^ vs. 33.16 m^3^) implied that the projected area influenced the accumulated sand volume. Nebkhas in site 1 tended to have large areas, low densities, and high spatial autocorrelation, while nebkhas in site 2 exhibited opposite trends with stochastic distribution. Mean vegetation density was significantly higher in site 1 than in site 2 (*p* < 0.05), while mean vegetation height exhibited an opposite trend (*p* < 0.05). In addition, there was higher vegetation coverage in site 1 than in site 2 (*p* > 0.05). According to the results, plant species (i.e., *N. tangutorum*) limited nebkha height under similar wind regimes regardless of the transport distance of aeolian material, while aeolian deposition and its effect on shrub growth jointly increased nebkha size.

## 1. Introduction

Nebkhas (coppice dunes) occur primarily in arid and semi-arid environments, such as depositional depressions in desertified steppes [1], margins of deserts [2], piedmont alluvial fans [3], and arable lands and grasslands in agro-pastoral transitional zones [4]. As plant-obstacle dunes, nebkhas are developed by the trapping of wind-borne sediment within or around the body of a plant [5]. Plants that exhibit resistance to drought, salinity, wind erosion, and sand burial stress grow normally and develop as the core structures of nebkhas [3,5,6]. The size and form of a nebkha depend on the size, density, and growth habit of the associated vegetation [1,7]. The presence of nebkhas facilitates plant recruitment and survival [8,9]. However, until a certain nebkha height is achieved, the efficiency of trapping sediment by the plant remains low [10,11] due to decreased sediment supply (interaction between sediment availability and wind-transport potential) and depressed plant growth under relatively poor soil water conditions [4,12,13]. Therefore, in the developing phase, nebkhas facilitate plant growth, while in the degradation phase, nebkhas impair and decrease plant growth [4,14,15]. Consequently, nebkha development could have both positive and negative feedback effects on plant development in arid environments.

In arid aeolian environments, plant species that form nebkhas are not readily killed by sand burial; instead, they grow vigorously via unique propagation mechanisms. Such plants species include shrubs such as *Tamarix* Linn. [3,6], *Karelinia caspia (Pall.)* Less. [16], *Ziziphus lotus* Blanco [1], and *Nitraria* L. [17,18,19]. To a large extent, the growth of shrubs based on their varying abilities to trap sand influences the morphology of nebkhas [6,7,17,20]. Consequently, competition between shrub growth and aeolian transport of sand material results in varying nebkha morphologies [21]. The heights, areas, and volumes of nebkhas increase with an increase in vegetation coverage [15]. Conversely, a decline in vegetation coverage and density increases the potential of soil erosion and the development of large nebkhas [8,22]. And the degree of aeolian sediment activity and supply can be estimated by the size of nebkhas and vegetation coverage. In addition, the greater sand volume in nebkhas under high vegetation densities than under low vegetation densities in shrub zones is due to higher sediment deposition within the plants [1]. The relationship between vegetation height and nebkha height is unclear, with positive [17] and no apparent [1] relationships reported previously. The development of nebkhas depends on the interaction between sand supply/mobility and vegetation growth. So, investigation of the effects of shrub growth on nebkha morphology could enhance our understanding of the influence of plant growth on nebkha development.

Some studies have reported that the sediment in nebkhas is generally derived from the adjacent topsoil and is transported only over a relatively short distance [7,17]. The availability of sand supply also depends on the degree of surface sand mobility and space between nebkhas. Studies on the spatial distribution of nebkhas can reveal the spatial heterogeneity and the intrinsic features of habitats [7]. And the spatial distribution of single nebkhas, in particular, indicates their relatively immobile and scattered forms in space [23]. The scale of spatially autocorrelation may play a pivotal role in nebkha development [24]. The small-scale spatial distribution patterns of nebkhas could reveal the underlying mechanisms driving large-scale ecological patterns and processes.

The plant component of an ecosystem is crucial, as it plays a significant role in preserving biodiversity [25], managing water resources [25], and facilitating the cycling of carbon and nitrogen [26,27]. *Nitraria tangutorum* Bobrov is widely distributed in desert ecosystems and plays a key role in the fixing and binding of mobile sand due to their drought tolerance, salt tolerance, and clonal propagation capacity via stems buried in sand, in addition to seed reproduction [14]. It grows in marginal regions of deserts, in inter-dune depressions, and in slightly salinized wetlands. Under natural reproduction conditions, *N. tangutorum* can trap sediment to form different sizes of nebkhas [28]. In the absence of human disturbance and long-term drought, *N. tangutroum* nebkhas can persist for long periods of time and, in turn, prevent the movement of sand [29]. Considering the importance of shrub canopy in nebkha formation and its feedback effects in ecosystem functioning [8,28,29,30], numerous studies have focused on the responses of shrub growth to wind–sand activity. Currently, only a few studies have explored the effects of shrub growth on nebkha development and spatial heterogeneity in arid environments in the case of *N. tangutroum*.

To better understand the associated relationship between nebkhas and shrub growth, two typical sites at the southeast margin of the Tengger Desert were selected (1) to analyze the effects of shrub growth on the morphology of nebkhas in the arid desert and (2) to explore factors influencing the spatial heterogeneity and pattern of nebkhas across different habitats.

## 2. Results

### 2.1. Morphological Features of N. tangutorum Nebkhas

**Figure 1:**
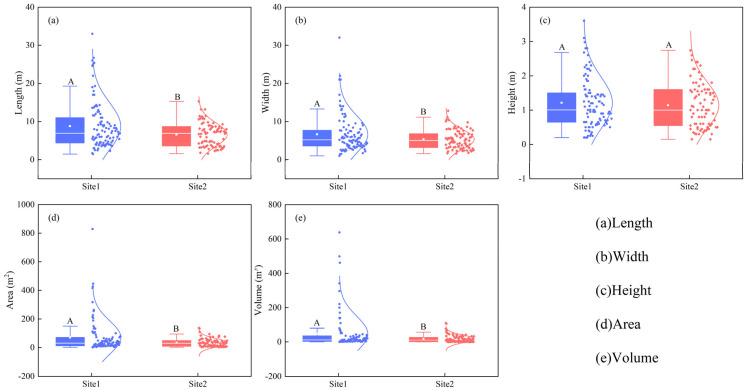
Morphological features of *Nitraria tangutorum* Bobrov nebkhas at the two study sites. Different capital letters in the figure indicate significant differences in morphological variables of nebkhas between two sites at *p* < 0.05.

Most of the *N. tangutorum* nebkhas observed in the study area had an ellipse mound shape. The mean length, width, projected area, and sand volume were all greater in nebkhas in site 1 (semi-fixed lake-basin lowland) than in site 2 (salinized fixed sand land; Figure 1a,b,d,e; *p* < 0.05), excluding height (Figure 1c; *p* > 0.05). For nebkhas occurring in site 1, the projected area ranged broadly from 1.43 to 829.16 m^2^, with a maximum height of 3.60 m (average, 1.2 m). For nebkhas in site 2, the projected area ranged from 2.01 to 137.3 m^2^, with a maximum height of 2.74 m (average, 1.14 m, Figure 1d).

The variations in all morphological features of nebkhas in site 1 were greater than the in site 2 (Figure 1). The frequencies of nebkhas with lengths < 14.0 m, widths < 10.0 m, and heights < 1.6 m were 86.3%, 83.3%, and 78.4%, respectively, in site 1, and 86.6%, 82.9%, and 79.3%, respectively, in site 2. The frequencies of projected areas < 100.0 m^2^ and sand volumes < 100.0 m^3^ were 81.4% and 88.2%, respectively, in site 1, whereas in site 2, more than 80% of the projected areas and sand volumes were below 50 m^2^ and 30.0 m^3^, respectively (Figure 1). The results showed that the horizontal and sand volume sizes of nebkhas were larger in site 1 than that site 2.

### 2.2. Spatial Distribution and Heterogeneity of N. tangutorum Nebkhas

**Figure 2:**
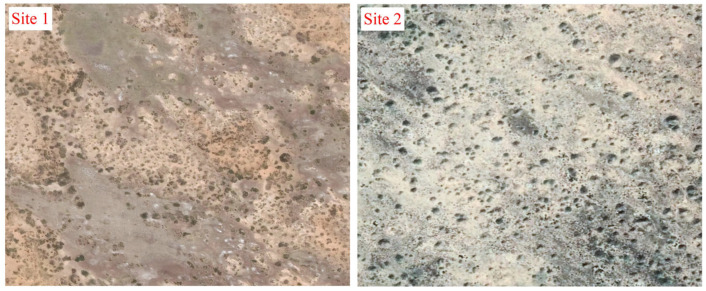
Spatial distribution pattern of *Nitraria tangutorum* Bobrov nebkhas (Google Earth images, Spatial resolution: ~0.13 m/pixel).

The spatial distribution of the nebkhas was aggregated in site 1 but dispersed in site 2 (Figure 2). All the morphological variables of nebkhas were tested to determine the variogram (Table 1). In site 1, there were local patterns of variation in all variables fitted to different models (spherical, exponential, or Gaussian). Nugget effects were very minor for all variables in the site (*C*_0_ = 0.00–0.14), indicating high spatial autocorrelation among the morphological variables. The autocorrelation scales of length, width, height, projected area, and sand volume of nebkhas were all <30 m. Within the range, the smaller the distance between single nebkhas, the stronger the spatial autocorrelation. High nugget to sill ratios (*C*/[*C*_0_ + *C*] > 75%) indicated that the spatial heterogeneity of all variables was primarily due to structural variance.

In site 2, the length, width, projected area, and sand volume of nebkhas were fitted to linear models. Based on the results of semi-variance analysis, nugget effects were very large, and the variation in those variables existed at a scale of more than 100 m and remained constant within the 100 m scale. Unlike the other four variables, the height of nebkhas fit the spherical model and was spatially autocorrelated within a range of 13.9 m. The ratio of nugget to sill for height (*C*/[*C*_0_ + *C*] < 75%) showed a moderate spatial dependence, indicating that the heterogeneity was caused by both random and structural variances.

Lower fractal dimensions for all variables in site 1 (*D* = 1.74–1.84) relative to site 2 (*D* = 1.94–1.99) also indicated higher autocorrelation in site 1 than in site 2 (Table 1).

### 2.3. Characteristics of N. tangutorum Shrubs

**Figure 3:**
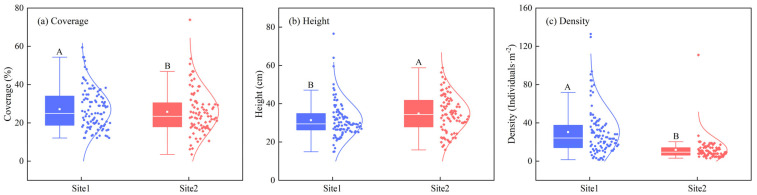
Characteristics of *Nitraria tangutorum* Bobrov shrubs. Different capital letters in Figure indicate significant differences in shrub characteristics between two sites at *p* < 0.05 (**a**) Coverage, (**b**) Height and (**c**) Density.

The mean shrub coverage and density of *N. tangutorum* in site 1 were greater than those in site 2; however, the mean shrub height exhibited an opposite trend (Figure 3). There were significant differences in shrub density and height between the two sites (*p* < 0.05), while no significant difference was observed in shrub coverage (*p* > 0.05). Variations in shrub coverage and density were greater in site 1 than in site 2, with an opposite trend observed for shrub height. Shrub coverage within the range of 10–40% was most prevalent across the two sites; the distribution of shrub coverage in this range was discrete in site 1 and centralized near the mean value in site 2. Shrub height was concentrated mostly within the 20–40 cm range in site 1, but was concentrated in the 20–50 cm range in site 2. Shrub density was mainly <40 individuals m^−2^ in site 1 and < 20 individuals m^−2^ in site 2 (Figure 3).

### 2.4. Relationships between N. tangutorum Nebkha Features and Shrub Characteristics

**Figure 4:**
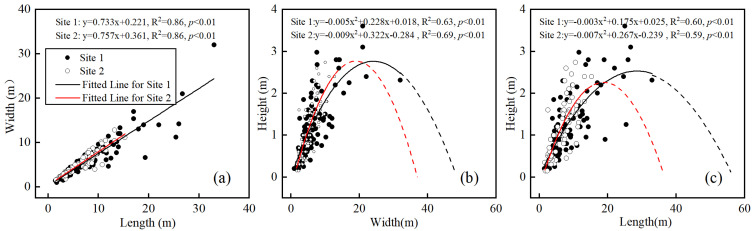
Scatter plots of nebkha sizes in the study sites. (**a**) Horizontal short axis (i.e., width) vs. vertical axis (i.e., height, sand burial depth), (**b**) horizontal long axis (i.e., length) vs. height, and (**c**) length vs. width. Black solid circles for site 1 and white empty circles for site 2. Solid line represents regression analysis and dot-dashed line indicates a prediction (Black line for site 1 and red line for site 2).

The lengths and widths of nebkha were synergistically enhanced (Figure 4a) and restricted by nebkha height (sand burial depth) (Figure 4b,c). There were no significant differences between the two sites in terms of threshold nebkha heights for width (2.62 and 2.60 m) and length (2.58 and 2.31 m, Figure 4b,c, *p* < 0.05). Corresponding to the threshold heights, the nebkha width and length in site 1 (22.80 and 29.17 m, respectively) were greater than those in site 2 (17.89 and 19.07 m, respectively). Between the two sites, there was a significant linear relationship between nebkha length and width (Figure 4a), in addition to a second-order polynomial correlation between length (width) and height (Figure 4b,c).

**Figure 5:**
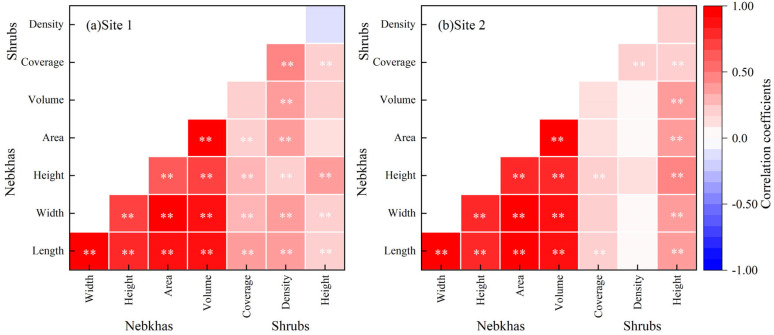
Correlation coefficients between variables of *Nitraria tangutorum* Bobrov shrubs and nebkhas. Length-horizontal long axis; width-horizontal short axis; height-vertical axis; area-projected area; volume-sand volume; coverage-shrub coverage; density-shrub density; height-shrub height. The asterisk indicates the level of significance: **—*p* < 0.01.

**Figure 6:**
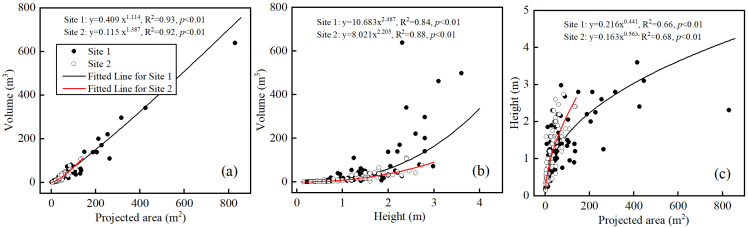
Relationship between accumulated sand volume and other morphological features of *Nitraria tangutorum* Bobrov nebkhas. (**a**) Sand volume vs. projected area; (**b**) sand volume vs. height (i.e., vertical axis, sand burial depth); and (**c**) height vs. projected area of single nebkha. Black solid circles for site 1 and white empty circles for site 2. Solid line represents regression analysis and dot-dashed line indicates a prediction (Black line for site 1 and red line for site 2).

Nebkha sand volumes were closely related to nebkha lengths, widths, heights, and projected areas (*p* < 0.01; Figure 5). Although projected areas and heights were power functions with sand volume, the effects of different project areas and heights on sand volume varied. In nebkhas with a specified sand volume, the larger the projected area was, the larger the sand volume would be. However, the effect of height on sand volume was mainly reflected in the greater height (Figure 6a,b). In small nebkhas, augmented projected area rapidly increased height, and the effect was weakened in larger nebkhas (Figure 6c).

**Figure 7:**
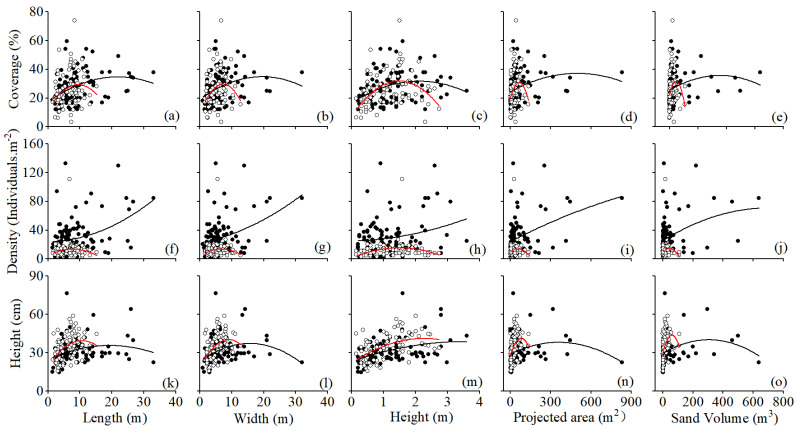
Relationships between features of *Nitraria tangutorum* Bobrov nebkhas (*x*-axis) and shrubs (*y*-axis). *X*-axis for nebkhas: length-horizontal long axis; width-horizontal short axis; height-vertical axis; area-projected area; volume-sand volume. *y*-axis for shrubs: coverage, density and height. (**a**–**o**) Black solid circles for site 1 and white empty circles for site 2. Solid line represents regression analysis and dot-dashed line indicates a prediction (Black line for site 1 and red line for site 2).

Both shrub density and height at site 1 were closely related to shrub coverage, while shrub height at site 2 was significantly correlated with coverage (*p* < 0.01; Figure 5). Nebkha sand volume had no correlation with vegetation coverage across both sites (*p* > 0.05); however, nebkha sand volume was significantly correlated with shrub density in site 1 and shrub height in site 2 (*p* < 0.01; Figure 5). Shrub coverage, density and height were restricted by nebkha length, width, height, projected area, and sand volume (Figure 7). The thresholds of shrub coverage and density values in site 1 were higher than those in site 2; however, the threshold of shrub height in site 1 was lower than that in site 2. Corresponding to the shrub thresholds, all nebkha morphological variables in site 1 were greater than those in site 2 (Figure 7).

## 3. Discussion

### 3.1. The Effect of Shrub Growth on the Morphological Features of Nebkhas

Across the two study sites in the Tengger Desert, *N. tangutorum* nebkhas were less than 3 m and had much lower heights (Figure 1) when compared with those observed in other regions, such as Kuwait, Western Mojave Desert, and the Molopo Basin in South Africa and Botswana, where nebkha height is usually above 3 m [9,31,32]. Our results are consistent with the features of nebkhas that develop in the marginal areas of shifting dunes, Gobi desert, grasslands, and formed around *Leguminosae* and *Ziziphus* shrubs [1,4,33], and *Tamarix* [3,32]. The changes in nebkha heights could be attributed to the differences in the responses of plant species to sand burial [6,7]. After sand burial, *N. tangutorum* maintained balance by increasing plant height or producing new branches [34,35,36]. *N. tangutorum* is a typical axial root plant with thick taproots and shallow roots [37]. Until a certain nebkha height is achieved, the efficiency of trapping sediment by the shrub remains low [10,11] due to decreased sediment supply (interaction between sediment availability and wind-transport potential) and depressed plant growth under relatively poor soil water conditions [4,12,13]. Different nebkha lengths and widths corresponded to specific nebkha height thresholds across the two sites (Figure 4b,c), reflecting that plant species play a role in restricting nebkha height.

As the sediment trapper, vegetation is a prerequisite for the emergence and development of nebkhas [38]. The dynamics of nebkhas are under the influence of feedback between deposition and vegetation growth [39]. Herein, we observed a second-order polynomial correlation between the shrub characteristics and morphological features of nebkhas across two sites (Figure 7a–o). The relationship implies that the positive effect of nebkha development in shrub growth was limited. After the nebkha variables corresponding to the thresholds of shrub growth were reached, further increases in nebkha length, width, height impaired shrub growth (Figure 7a–o). Our finding corroborated the conclusion of with regard to the relationship between nebkha development and shrub growth in Alaxa Plateau and in semi-arid northern China [4,10]. Together, these findings suggest that nebkha development could inhibit *N. tangutorum* shrub growth (Figure 7a–o) and even lead to nebkha degradation to a certain extent. Such degradation could be reflected in the increased length and width in addition to decreased height in nebkhas (Figure 4b-c).

Shrub coverage is one of the key factors influencing wind erosion and sediment processes [20,22]. In our study, differences in shrub density and height were significant between two sites but no differences were observed in terms of shrub coverage (Figure 1 and Figure 3), indicating that the growth of *N. tangutorum* had the different adaption mode to sand burial across different habitats. The increased shrub density and height had the same effect as the coverage, which had a positive effect on the trapping sand sediment [3,40]. Our result is inconsistent with the findings of Tengberg and Chen reported for *Z. lotus* nebkhas in Tunisia [1]. Although *N. tangutorum* has a prostrate growth habit and its branches tend to produce adventitious roots after sand burial [14], the branches could maintain an upright growth position by minimizing aeolian erosion [32,41].

Across the two sites, higher threshold values of shrub height were associated with smaller morphological variables of the *N. tangutorum* nebkha (Figure 7k–o). But the higher threshold of shrub density (Figure 7 f–j) and coverage (Figure 7a–e) corresponded to the larger variables of nebkhas. Differences in shrub growth formed the large-size nebkhas in the semifixed site and small-size nebkhas in the fixed site, indicating the effect of shrub growth on formation of nebkhas consistent with results between nebkha development and shrub coverage from coastal dune-fields of the arid region [7]. The threshold shrub coverage was nearly or < 30% in both sites regardless of nebkha size (Figure 6c), which was consistent with the finding of a prior study conducted in southwest Kalahari Desert [22]. The role of shrub coverage in promoting nebkha development changes with shifts in nebkha surface activity [22,42]. However, moderate surface activity can stabilize active dunes through stimulated shrub colonization processes in the semi-fixed lake-basin lowland site [43,44]. This could also be attributed to shallow groundwater, which provided a favorable environment [22]. Therefore, shrub coverage could control not only nebkha height but also nebkha length and width. The similar threshold of shrub coverage in relation to different heights of nebkhas across the two study sites (Figure 6c) revealed that plant growth could influence the morphology of nebkhas [3,45,46,47,48]. The effect is mainly reflected in the responses of the nebkhas in their horizontal sizes to shrub density and height, in addition to shrub coverage under different environments. The measured morphological variables of *N. tangutorum* nebkhas were higher in the semi-fixed lake-basin lowland than in the salinized fixed sand site (Figure 7).

### 3.2. Possible Factors Affecting the Morphology and Spatial Heterogeneity of Nebkhas

Nebkhas occurring in different habitats vary in size depending on vegetation development, wind regime, sediment availability, and other factors such as anthropogenic activities and climate change [1,6,49]. Consistent with prior studies [5], our results showed that there were high correlations between the morphological variables of *N. tangutorum* nebkhas (Figure 6). A second-order polynomial correlation between the height (sand burial depth) and length (or width) of nebkhas (Figure 4b,c) suggests that the scale of nebkha development was limited by nebkha height (sand burial depth) across the two study sites in the Tengger Desert [1,33,50], and that the nebkhas evaluated were mostly still growing [1,33]. The high soil moisture beneath nebkhas across two sites [51] provided vital water resources for shrub growth in dry period. Augmented projected area had a major contribution to the increase in height of small nebkhas (i.e., early stages), while the effect weakened in large nebkhas (i.e late stages) (Figure 6c). The expansion and merging of adjacent nebkhas increased the horizontal scale due to height limitation of nebkhas, but the effect of merging on the vertical scale is much smaller than that of the horizontal scale (Figure 1). And the aforementioned changes may result in more mobilized sand and further benefit to horizontal expansion of nebkhas [23].

**Figure 8:**
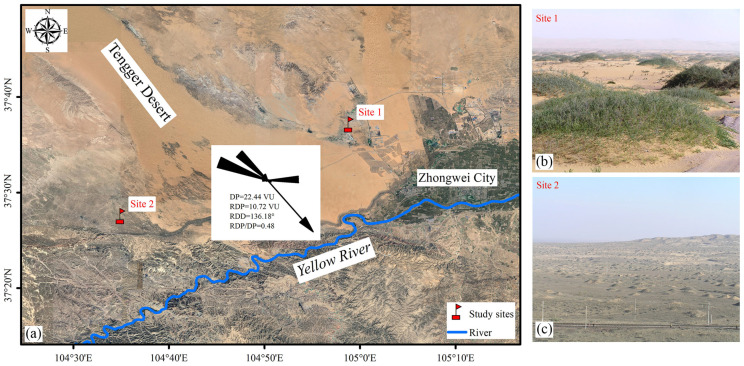
(**a**) Location of two study sites in the Tengger Desert, Northern China. (**b**) Site 1 is a semi-fixed desert lake-basin lowland habitat and (**c**) site 2 is a salinized fixed sand land habitat.

In addition to plant species and its growth level, the origin and development of nebkhas depends on the migration and accumulation of sediment across distant and adjacent regions. However, the sediment in nebkhas is generally derived from the adjacent topsoil and, therefore, has been transported only over short distances [4,47,52]. Nebkhas are primarily formed through local redistribution of sediment from neighboring interdune areas by creep and saltation of sediment material [12,13]. Herein, based on the different geographic locations and degree of sand-fixation between the two study sites, the semi-fixed lake-basin lowland site (site 1) in the desert heartland had higher sediment availability and mobility than the salinized fixed sand site (site 2). Therefore, larger *N. tangutorum* nebkhas (projected area) developed in the semi-fixed lake-basin lowland; in contrast, nebkhas tended to be singular and small in the salinized fixed sand site (Figure 8). This phenomenon is consistent with the development of foredunes in coastal dune fields [7,12,21,43]. The sediment in adjacent nebkhas may increase the capacity of nebkhas to accumulate sand material by stimulating shrub growth [14,53]. According to Cooke [5] the cores of nebkhas grow to a height at which wind velocity can re-entrain sediment. In our study area, only the topsoil contained loose sandy sediment [51] due to the limitation of groundwater and soil water for shrub growth [22,54]. When the topsoil is lost, therefore, the main source of sediment for nebkhas is lost. Subsequently, wind flow becomes undersaturated and soil erosion takes place [54]. In such cases, nebkhas exceeding a threshold level of vertical development causes the projected area to increase because of the breakdown or emergence of adjacent nebkhas [43]. Thus, the increase in sediment supply and mobility in adjacent nebkhas may improve the capacity of nebkhas to accumulate sand material by stimulating shrub growth [14,53]. Therefore, adjacent nebkhas sizes are correlated (i.e., spatial autocorrelation), and an increase in nebkha size is reflected in an increase in horizontal scale [24,43].

The aggregated distribution of nebkhas in the semi-fixed lake-basin lowland and dispersed distribution in the salinized fixed sand site (Figure 2) were also verified by the role of adjacent nebkhas based on the analysis of Geo-statistics (Table 1). All morphological variables showed high spatial autocorrelation in the semi-fixed lake-basin lowland within 30 m (Table 1). In contrast, constant variances in length, width, projected area, and sand volume within 100 m and spatial autocorrelation in height within 13.9 m were observed in *N. tangutorum* nebkhas in the salinized fixed sand site (Table 1). The spatial heterogeneity of nebkhas’ morphology relied on structure factors such as their length, width and height in a semi-fixed site and constant variances resulted from stochastic factors [55] in the salinized fixed sand site. Then, high spatial organization of shrub and nebkha in a limited scale could form the aggregated distribution pattern, but stochastic factors such as sand supply or activity, state of soil water, and soil crusts development for an improved colonization chance for seeds, seedling survival and growth resulted in a dispersed distribution with a greater density of nebkhas [7,33].

## 4. Materials and Methods

### 4.1. Site Description

The study area is a typical transition zone between steppe desert and desert in Northern China, and the altitude varies between 1200 and 1400 m [51]. The area is characterized by a temperate continental arid climate. The mean annual temperature is 10 °C and the effective accumulated temperature is 3000 °C or more. The mean annual precipitation is 180.2 mm with large interannual variability, 80% of which falls between mid-June and mid-September. Using the wind speed and direction data during May 2021 and April 2022 in Zhongwei City, China, the drift potentials (DPs) in the study area were calculated by Fryberger’s formula [56]. Sand-moving wind at speeds greater than 6 m/s accounted for 3.94% of the whole year. The annual DP was 22.44 VU, which indicates a low wind energy environment according to Fryberger’s categorization standard of wind energy environment (Figure 1a). The DPs in the WNN-NW and E directions were dominant, accounting for 63.43% and 21.52%, respectively. The resultant drift potential (RDP) was 10.72 VU and the resultant drift direction (RDD) was 136.18°. The annual RDP/DP, an index of the directional variability of the wind, was 0.48. The DP was high in Spring (March to May), accounting for 60.29% of the annual DP.

*N. tangutorum* nebkhas mainly occur in the marginal areas of the Tengger Desert, such as lake-basin oases and the lower parts of the low-mountain alluvial plain, with a groundwater table level of 1.5–4 m [57]. The natural vegetation in the study area consists of shrubs, mostly *N. tangutorum*, which is often associated with nebkha development [57]. *N. tangutorum* nebkhas are composed of aeolian sand, and the underlying strata are mostly alluvial and lacustrine sandy soil, which are clayey and slightly salinized. The grain size of nebkhas is dominated by fine sand (>80%), similar to that of the adjacent mobile dunes [51]. Two representative sites were selected in the study area based on the soil types of the underlying strata associated with *N. tangutorum* shrubs (Figure 8). The specific parameters of soil physical and chemical properties have been identified in [58].

The types of mobile dunes in the study area were mainly crescent dune chains and reticulate dunes, etc. Site 1 was a salinized wetland habitat in the semi-fixed lake-basin lowland with a shallow groundwater table (1–2 m). Site 1 is surrounded by mobile sand dunes, and the closest distance to the mobile sand dune areas is 1.5 km. The soil type of the underlying strata was solonchaks. Due to the influence of the Tengger Desert, some places were covered by sand or dunes, and saline soil crusts occur on the surfaces of the internebkha spaces. Dwarf shrub (*Kalidium foliatum* (Pall.) Moq.) with 2–3% coverage occasionally occur in the internebkha lowlands. In the presence of *N. tangutorum* shrubs, nebkhas are formed by aeolian sand at a semi-fixed state. Site 2 was a salinized fixed sand land on the piedmont sand-covered alluvial fan. The mobile sand dunes are mainly distributed on the east side of Site 2, and the closest distance to the mobile dune area is about 4 km. The soil type of the underlying strata was fluvisols and the physiognomy features alternated between nebkhas and clay interdune depressions. The groundwater table was deep (3–5 m or more). There were thin and fragile lichen (*Collema tenax* [Sw.] Ach) crusts on the surface of *N. tangutorum* nebkhas in fixed states. Internebkha spaces were covered by sand or thin layers of sand, which had desalted and were alkaline in pH. The annual herbs with 3–5% coverage on the internebkha lowland consisted of *Corispermum* spp., *Bassia dasyphylla* (Fisch. & C.A.Mey.) Kuntze, *Stipa glareosa* P.A. Smirn., *Cleistogenes songorica* (Roshev.) Ohwi, and *Echinops davuricus* Fisch. ex Hornem [57].

### 4.2. Study Design

A flat plot (100 × 100 m) was selected in each of the two study sites. In each plot, the morphometric variables of 102 *N. tangutorum* nebkhas (site 1) and 82 *N. tangutorum* nebkhas (site 2) were measured in August, 2004. The horizontal long axis (*L*, length), the horizontal short axis (*W*, width), and the vertical axis (*H*, height, i.e., the sand burial depth) of each nebkha were measured. Briefly, the height between the zenith and the ground was measured as the sand burial depth. Areas (*A*, projected area) of single nebkhas were calculated using the formula for calculating the area of an ellipse, *A* = [π(*L* × *W*)]/4 [9]. Volumes (*V*, sand volume) of single nebkhas were calculated using half the volume of an ellipsoid, *V* = [(4A × H)/3]/2. To determine the locations of nebkhas, an angle of the sample plot was taken as the point at which its *x* and *y* coordinates were (0,0), and the coordinates of the central point of the cross line in each nebkha were determined relative to the origin in the sample plot [55]. In addition, three quadrats (1 × 1 m) were selected on each nebkha to survey the height, coverage, and density of *N. tangutorum* shrubs. The shrub density was estimated by a single branch number method. The shrub height was the natural height of each branch.

### 4.3. Data Analysis

The spatial variability of *N. tangutorum* nebkhas was analyzed and explained based on the results obtained following the calculation of a variogram $\gamma \left(h\right)$ as follows [59,60]:

$$\gamma \left(h\right)=\frac{1}{2N\left(h\right)}\sum_{i=1}^{N\left(h\right)}{\left[Z\left(\mathrm{xi}\right)-Z\left(\mathrm{xi}+h\right)\right]}^{2}$$

where $N\left(h\right)$ is the number of pairs of sampling points separated by the distance *h*, $Z\left(\mathrm{xi}\right)$ is the value at the distance xi, and $Z\left(\mathrm{xi}+h\right)$ is the value at the distance xi+h. Several key parameters can be obtained based on the variogram and its curve:

① With an increase in the spatial distance *h*, the variogram varies from a non-zero value to a stable constant, i.e., sill value (*C_o_ + C*); ② when *h* = 0, γ(0) = *C_o_*, i.e., nugget value; and ③ when $\gamma \left(h\right)$ reaches the sill’s spacing (range) and fractal dimension *D*, the *D* value can be determined based on the relationship between $\gamma \left(h\right)$ and *h*, $2\gamma \left(h\right)=h^{\left(4-2D\right)}$ [60]. The larger the *D* value, the higher the proportion of spatial heterogeneity caused by random factors. The spatial heterogeneity was determined by comparing the D values (dimensionless numbers) of different variables [59].

The data were processed and analyzed using IBM SPSS Statistics 24 (IBM Corp., Armonk, NY, USA) and GS^+®^ Version 5 (Gamma Design Software LLC., Plainwell, MI, USA). Between-site differences in the morphological features of nebkhas and vegetation characteristics of *N. tangutorum* shrubs were tested using one-way ANOVA. The least significant differences test was used for multiple comparisons, while Pearson’s test was used to determine correlations between different variables of *N. tangutorum* nebkhas and shrubs (*p* < 0.05 and *p* < 0.01). All the morphological variables of nebkhas were tested for normality using the Kolmogorov–Smirnov test.

## 5. Conclusions

Our study indicated that the positive interaction between nebkhas development and shrub growth has a threshold. The similar thresholds of vertical development regardless of the horizontal size of nebkhas indicated that plant species limited the vertical development of nebkhas under similar wind regimes. The higher thresholds of shrub coverage and density corresponded to greater sizes of nebkhas in semi-fixed lake-basin lowland than that of the salinized fixed sand, combined with higher thresholds of shrub height in accordance with smaller-sized nebkhas, motivated the formation of larger-sized nebkhas in a semi-fixed site. The results implied that the semi-fixed lake-basin lowland offered a more favorable environment for *N. tangutorum* shrub growth. Differences in the nebkha features reflected the physiological and ecological adaptations of *N. tangutorum* shrubs to environmental conditions such as high sand mobility and supply for growth of shrub density and low sand mobility and supply for shrub height. The high spatial autocorrelation of nebkhas resulted in aggregated distribution in semi-fixed lowland and high stochastic factors formed the dispersed distribution of nebkhas in the salinized fixed-sand site.

The degree of sand-fixation and sand supply/mobility had a major influence only on the horizontal variables (i.e., length, width, and projected area) of *N. tangutorum* nebkhas, while plant species and growth were the major factor influencing the vertical nebkha development (i.e., sand burial depth) under similar wind regimes. The groundwater level may also be an important factor to promote nebkha development by maintaining shrub growth in dry periods. The analysis of the factors affecting the spatial heterogeneity of nebkha variables provided a basis for biological sand control in arid regions. Additionally, the results contribute to a deep understanding of the relationship between landscape change and vegetation development.

## Figures and Tables

**Table 1:** Semi-variogram model parameters of morphological variables of *Nitraria tangutorum* Bobrov nebkhas.

Site	Variable	Model	*C*_0_	*C*_0_ + C	Effective Range/m	*C*/*C*_0_ + *C*	*D*	Distribution	Transformation Model
1	Length	Spherical	0.01	0.07	26.90	0.85	1.82	Normal	—
	Width	Exponential	0.00	0.07	27.00	0.97	1.84	Normal	—
	Height	Gaussian	0.08	0.60	19.05	0.88	1.74	Normal	—
	Area	Spherical	0.05	0.28	26.90	0.84	1.82	Non-normal	Napierian logarithm
	Volume	Spherical	0.14	0.61	29.30	0.78	1.83	Non-normal	Napierian logarithm
2	Length	Linear	10.06	10.06	66.75	0	1.96	Normal	—
	Width	Linear	6.51	6.51	66.75	0	1.99	Normal	—
	Height	Spherical	0.13	0.42	13.9	0.70	1.94	Normal	—
	Area	Linear	902.47	902.47	66.75	0	1.99	Normal	—
	Volume	Linear	0.48	0.48	66.75	0	1.99	Non-normal	Napierian logarithm

Length-Horizontal long axis; Width-Horizontal short axis; Height-Vertical axis; Area-Projected area; and Volume-Accumulated sand volume of single nebkhas. *C*_0_-Nugget variance; *C*_0_ + *C*-Structural variance sill; and *D*-fractal dimension.

## Data Availability

Privacy issues are present, data should not be shared.
